# 

**Published:** 1885-11

**Authors:** 


### CONTENTS.

ORIGINAL ARTICLES—Some Remarks on the Dengue Epidemic—Carbolic Acid Injections in the Treatment of Carbuncle—A Case of Hemorrhage Before Delivery .......... 205–220

CULLINGS FROM CONTEMPORARIES—The Intelligent care of Dumb Brutes—Vaccination as a Remedy for Whooping-Cough—Dielectrolysis—A New Hæmostatic .......... 222–226

CORRESPONDENCE—Declines the Honor—Our New York Letter .......... 227–228

EDITORIAL—Is it Dengue—Sauce for the Goose—The Micrococcus of Dengue—Gush — Delinquents to Texas State Medical Association — Small but Select .......... 233–243

BIBLIOGRAPHY—Books Received .......... 244

MELANGE—Sorry Birds—Our Turn — An Emulsion—Journal Changes .......... 245–246

ADVERTISERS' NOTICES — Acid Phosphate — The Sick Baby—Drs. Pope and Hall—The Microscope in Texas—Digestive Ferments .......... 246–248

